# Above and Beyond Robotic Surgery and 3D Modelling in Paediatric Cancer Surgery

**DOI:** 10.3389/fped.2021.777840

**Published:** 2021-12-20

**Authors:** Laura Privitera, Irene Paraboschi, Kate Cross, Stefano Giuliani

**Affiliations:** ^1^Wellcome/Engineering and Physical Sciences Research Council Centre for Interventional & Surgical Sciences, University College London, London, United Kingdom; ^2^Developmental Biology and Cancer Department, University College London Great Ormond Street Institute of Child Health, London, United Kingdom; ^3^Department of Specialist Neonatal and Paediatric Surgery, Great Ormond Street Hospital for Children NHS Foundation Trust, London, United Kingdom

**Keywords:** paediatric surgery, oncology surgery, optical imaging, spectroscopy, cancer imaging, novel intraoperative technologies, fluorescence-guided surgery, children

## Abstract

Although the survival rates for children's cancers have more than doubled in the last few decades, the surgical practise has not significantly changed. Among the most recent innovations introduced in the clinic, robotic surgery and augmented reality are two of the most promising, even if they are not widespread. The increased flexibility of the motion, the magnification of the surgical field and the tremor reduction provided by robotic surgery have been beneficial to perform complex oncological procedures in children. Besides, augmented reality has been proven helpful in planning for tumour removal, facilitating early discrimination between cancer and healthy organs. Nowadays, research in the field of surgical oncology is moving fast, and new technologies and innovations wich will help to shape a new way to perform cancer surgery. Paediatric surgeons need to be ready to adopt these novel devices and intraoperative techniques to allow more radical tumour resections with fewer complications. This review aims to present the mechanism of action and indications of several novel technologies such as optical imaging surgery, high definition cameras, and intraoperative loco-regional treatments. We hope this will enhance early adoption and more research on how to employ technology for the benefit of children.

## Introduction

With 367,000 new cases in the UK every year, paediatric and adult solid cancers are among the top causes of death worldwide. Although survival rates for children's cancers have more than doubled between the 1970s and 2000s, oncology surgery has not significantly changed in the last thirty years (1).

The surgical resection of tumours still represents one of the main treatments for nearly all the new solid cancer diagnosis (1). According to the literature, clear surgical margins and a maximal degree of tumour resection strongly impact patients' outcomes (2, 3). However, the delineation of cancer margins and the microscopical clearance of the disease remain significant challenges. One of the techniques more recently adopted to enhance live surgical precision and accuracy during complex oncological procedures is robotic surgery (Table 1) (4, 6, 10, 16–20). The increased flexibility of the motion, the surgical field's magnification with enhanced 3D visualisation, and the tremor reduction have all been described as particularly useful to perform even challenging surgical procedures in children. Besides, the creation of 3D models in preparation for surgery or superimposed onto the surgical field have also been trialled to enhance the surgical practise (21–25). Furthermore, augmented reality acts as an effective adjunct by increasing peri-operative information, and it has been proven beneficial when removing tumours, facilitating discrimination between malignant tissue and adherent healthy organs (22, 26–28).

**Table 1:** Current literature focusing on robotic surgery used for paediatric oncology surgery.

**References**	**Type of study**	**Number of patients**	**Mean age (years)**	**Pathologies included**	**Mean total operative time**	**Conversion rate**	**Mean days of hospitalisation**	**Robotic system**
Blanc et al. (4)	Prospective observational study	89	8.2	Neuroblastomas (*n* = 18) Ganglioneuroblastomas (*n* = 4) Ganglioneuromas (*n* = 9) Wilms' tumours (*n* = 20) Neuroendocrine tumours (*n* = 12) Adrenal tumours (*n* = 9) Germ-cell tumours (*n* = 7) Pancreatic tumours (*n* = 4) Thymic tumours (*n* = 4) Inflammatory myofibroblastic (*n* = 4) Different rare tumours (*n* = 5)	215 min	8%	3	NA
Meehan et al. (5)	Retrospective study	14	NA	Neuroblastoma (*n* = 3) Ovarian teratoma (*n* = 1) Abdominal lymphangioma (*n* = 1) Retroperitoneal tumour (*n* = 1) Pancreatic tumour (*n* = 1) Mediastinal germ cell tumour (*n* = 1) Mediastinal teratoma (*n* = 1) Posterior mediastinal mass (*n* = 2) Not specified abdominal tumour (*n* = 1) Pheochromocytoma (*n* = 1) Mediastinal inflammatory mass (*n* = 1)	NA	29%	NA	Da Vinci system (Intuitive Surgical, Sunnyvale, CA)
Meignan et al. (6)	Multicenter retrospective study	11	7.65	Nephroblastoma (*n* = 1) Metanephric adenoma (*n* = 1) Neuroblastomas (*n* = 3) Pheochromocytomas (*n* = 2) Adrenocortical adenomas (*n* = 2) Cystic lymphangioma (*n* = 1) Paraganglioma (*n* = 1) Pancreatic cyst (*n* = 1)	145 min	8.3%	3	Da Vinci system (Intuitive Surgical, Sunnyvale, CA)
Blanc et al. (7)	Prospective study	10	5	Wilms tumour (*n* = 8) Renal sarcoma (*n* = 1) Renal tubulopapillary carcinoma (*n* = 1)	270 min	30%	5.6	Da Vinci system (Intuitive Surgical, Sunnyvale, CA)
Varda et al. (8)	Retrospective study	8	12.5	Papillary renal cell carcinoma (*n* = 1) Segmental cystic dysplasia (*n* = 2) Benign heterologous tissue with nephrogenic rests (*n* = 1) Embryonal non-seminomatous germ cell tumour (*n* = 1) Rhabdomyosarcoma (*n* = 1) Ganglioneuroma (*n* = 1) Unclassified spindle cell sarcoma (*n* = 1)	277 min (PN) 540 min (RPLND)	0	3.7	NA
Meehan et al. (9)	Case reports	5	9.8	Ganglioneuroma (*n* = 1) Ganglioneuroblastoma (*n* = 1) Teratoma (*n* = 1) Germ cell tumour (*n* = 1) Large inflammatory mass (*n* = 1)	113 min	0	1.4	Da Vinci system (Intuitive Surgical, Sunnyvale, CA)
Navarrete Arellano and Garibay González (10)	Prospective, observational, longitudinal study	4	4.7	Mediastinal teratoma (*n* = 1) Retroperitoneal lipoma (*n* = 1) Pheochromocytoma (*n* = 1) Not specified (*n* = 1)	NA	NA	NA	Da Vinci system (Intuitive Surgical, Sunnyvale, CA)
Cost et al. (11)	Case report	1	14	Renal cell carcinoma	180 min	0	2	Da Vinci system (Intuitive Surgical, Sunnyvale, CA)
Hassan et al. (12)	Case report	1	16	Left ventricular myxoma	NA	0	3	Da Vinci system (Intuitive Surgical, Sunnyvale, CA)
Akar et al. (13)	Case report	1	15	Cystic adenomyoma	NA	0	NA	NA
Backes et al. (14)	Case report	1	18	Mullerian rhabdomyosarcoma	315 min	0	5	NA
Anderberg et al. (15)	Case report	1	1.8	Embryonal rhabdomyosarcoma	Min	0	NA	Da Vinci system (Intuitive Surgical, Sunnyvale, CA)

*NA, not available; PN, partial nephrectomy; RPLND, retroperitoneal lymph node dissection*.

However, beyond the applications of robotic surgery and augmented reality, there has been a significant drive toward the clinical translation of other technologies, devices and intra-operative treatments to enable surgeons to perform safer and more radical resections. We envisage that optical imaging surgery, high definition cameras and intraoperative loco-regional therapies have the potential to revolutionise surgical oncology through more effective visualisation and removal of cancer. The aim of this article is, therefore, to review these novel promising technologies and devices to disseminate their application and facilitate a quicker adoption in the field of paediatric surgery.

## Optical Imaging in Surgery

**Figure 1:**
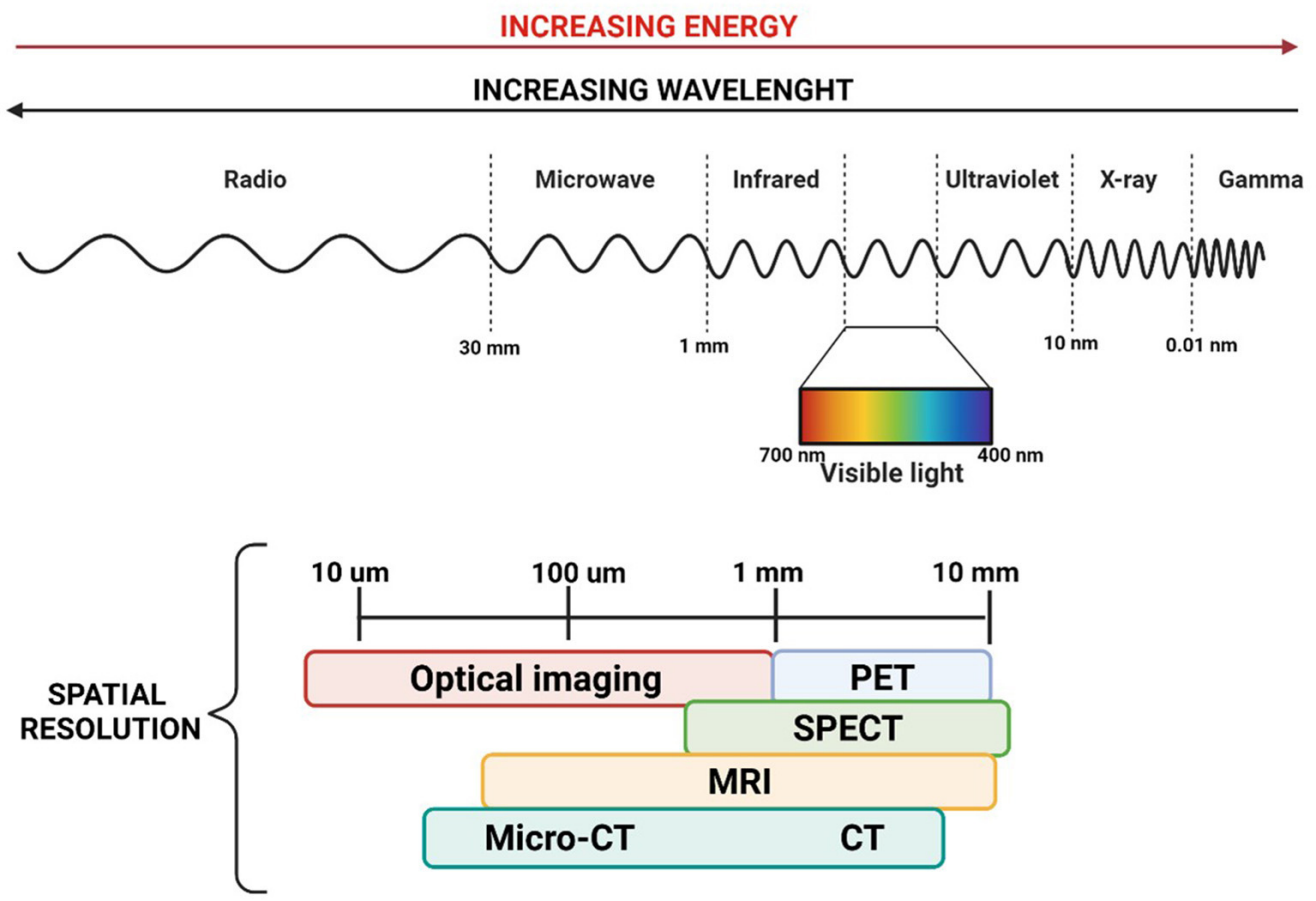
Image of the electromagnetic spectrum and the associated wavelength and energies, with a focus on the spatial resolution of the dominant medical imaging modalities. TC, computer tomography; MRI, magnetic resonance imaging; SPECT, single-photon emission computed tomography; PET, positron emission tomography.

One of the crucial aspects of performing safe and effective cancer removal is visualising normal anatomical structures and differentiating them from the disease. Although human eyes can reconstruct the shape and architectural features, they cannot distinguish between spectra with slight separation in wavelengths, meaning that different tissues are very similar in colour (29). MRI, CT, ultrasonography, and nuclear imaging scans play an important role in the pre-operative assessment, allowing high-resolution whole-body imaging. However, their use in the operative field is limited (30, 31). There has been recently increased interest in the clinical application of optical imaging techniques in the operating theatre. Optical imaging offers a detailed picture of body anatomy, offering high-resolution images of entire organs down to molecules smaller than 10 μm, using non-ionising radiation, including visible, ultraviolet and infrared light (Figure 1) (29, 32).

### Fluorescence-Guided Surgery and Dye-Loaded Targeted Probes in Cancer Surgery

One of the most commonly used optical imaging techniques is fluorescence-guided surgery (FGS). FGS principle is to generate a real-time fluorescence image of the surgical region to help the surgeon delineate their targets (33). Compared to other intra-operative devices, fluorescence imaging systems are relatively affordable, and they do not require specific training as they are generally intuitive to use. The key elements of FGS are a contrast agent (usually administered before the procedure), a light source, philtres for the excitation of the fluorescence agent and a camera to detect the signal designed for either laparoscopic, open or robotic procedures.

Near-infrared (NIR) fluorescence imaging provides a new and versatile platform for visualisation, resection and treatment. Imaging systems are often calibrated around the excitation and emission spectrum of indocyanine green (ICG), widely applied in surgery. In the paediatric field, ICG has been used safely and efficiently to delineate the biliary flow during laparoscopic cholecystectomies, assess bowel perfusion in neonates with necrotising enterocolitis, map arteries during urogenital surgeries and reconstruct the microvascularity in plastic surgery (34, 35). However, it is mainly in the oncological field that FGS has been emerging as a cutting-edge innovation. The live visualisation of the 3D tumour anatomy can facilitate the differentiation between the tumour and the normal tissue and lead to more radical tumour resections with improved surgical outcomes (34, 35). ICG-optical imaging has been adopted for the surgical treatment of several paediatric cancers, including hepatoblastomas, osteosarcomas, non-rhabdomyosarcomas, rhabdomyosarcomas, neuroblastomas, Ewing sarcoma, germ cells tumours, chondroblastoma, solid pseudopapillary neoplasms of the pancreas, lymphoma, and myoepithelial carcinoma (34, 35). Although ICG navigation imaging for paediatric hepatoblastomas is at its initial stages, early results seemed to be very promising (34–39). In particular, ICG-angiography has been very helpful to detect the primary tumour and its peritoneal and lung metastases (36, 36–39). Despite these encouraging results, some issues such as the background noise from adjacent organs, the tissue attenuation and the limitation in-depth penetration still must be addressed (35). Shortly, the record of more cases will lead to more standardised procedural protocols for establishing the optimal timing and dosage for ICG injection and a more accurate patients' selection. Further developments in optical imaging technology will soon overcome these limits, providing fluorescent dyes and detection cameras with greater tissue penetration.

Other than ICG, several reports, critical and systematic reviews have discussed the role of 5-Aminolevulinic acid (5-ALA)-guided surgery in performing a gross total resection of brain tumours in children, with the ultimate aim to improve patients' survival and reduce the risk of recurrences (40–43). The extent of surgical resection is a strong prognostic factor in paediatric brain tumours. However, performing a complete tumour excision can be a real challenge, as these tumours frequently infiltrate adjacent vulnerable tissue, making their clear intraoperative identification particularly demanding. In this regard, the literature seems to support the role of 5-ALA-guided resection to identify brain tumours more efficiently, especially in the case of glioblastoma, anaplastic ependymoma WHO grade III and anaplastic astrocytoma (43). Due to the very promising results achieved by these early studies, the significant impact of this new approach and its less clear role in infratentorial tumours, prospective randomised clinical trials have been advocated to increase the overall level of evidence concerning the usage of 5-ALA in the paediatric population (40–43).

Although this decade has undoubtedly witnessed significant advances in the clinical application and technical development of ICG and 5-ALA optical imaging, there is still room for further developments. For example, tumour-specific targeted probes are currently under investigation to maximise the tumour signal and minimise background noise. This is of particular interest in the paediatric population, as there are tumour-specific monoclonal antibodies that have been already clinically approved (34, 44). The first preclinical study on a molecular-targeted fluorescent agent for FGS in paediatric oncology was provided by Wellens et al. (27) who developed and evaluated a GD2-specific tracer consisting of the immunotherapeutic antibody Dinutuximab-beta, conjugated to the NIR-I fluorescent dye (IRDye800CW). Their results showed the specific binding of anti-GD2-IRDye800CW to human neuroblastoma (NB) cells both *in vitro* and *in vivo* models. FGS using a tumour-specific tracer in paediatric oncology surgery remains experimental but is a promising modality for localising tumours and their metastases and protecting peritumoral organs and vital structures (34). The development of novel fluorescent probes and the identification of new tumour targets gives the surgeon the perfect combination to enhance cancer surgery, particularly for solid tumours. There is an excellent potential for this methodology to enter routinely in the surgical setting. As such, surgeons needs to familiarise themself with the basic pharmacokinetics of these novel molecules and the devices to detect them intraoperatively.

### Spectroscopy-Guided Surgery

**Figure 2:**
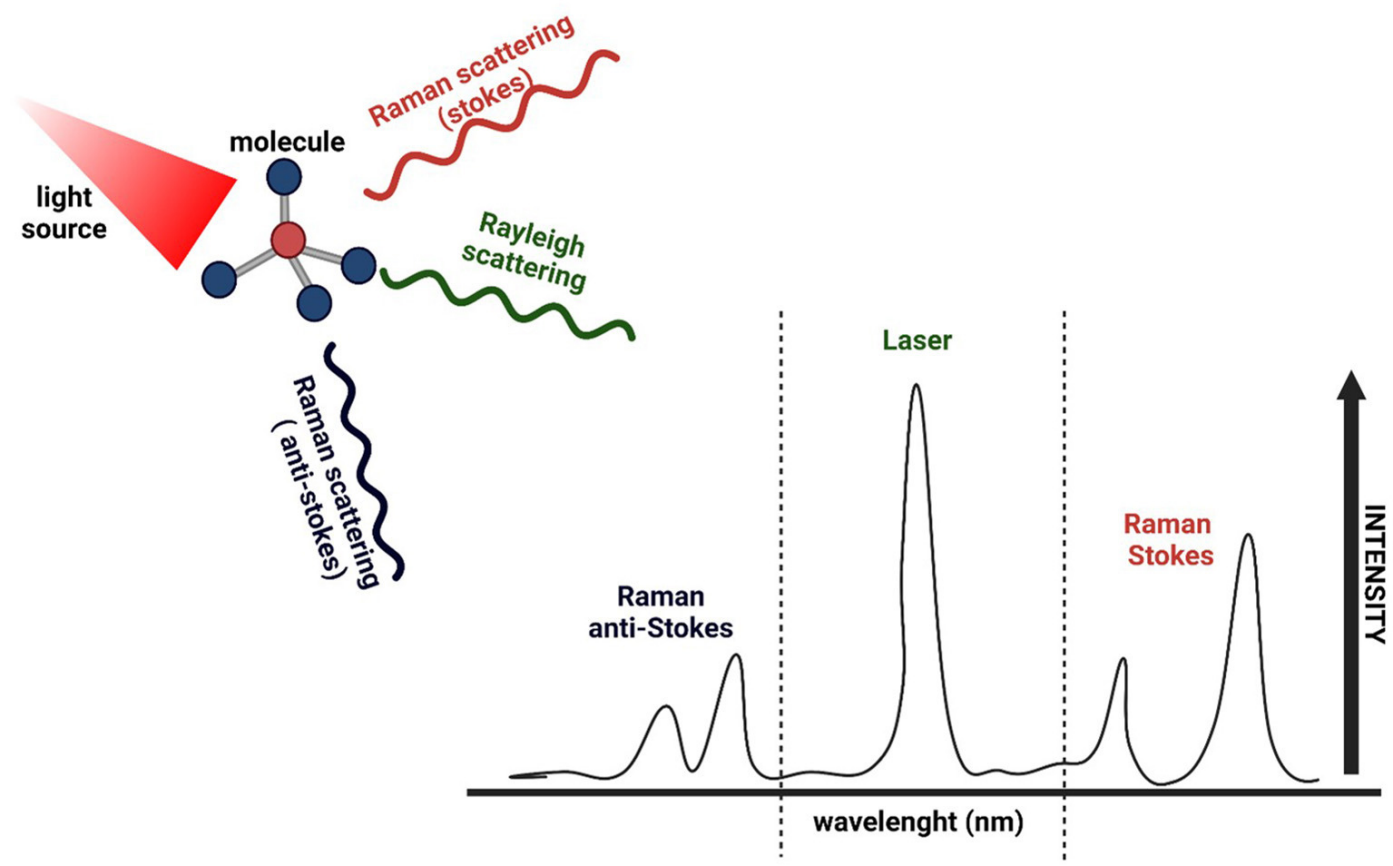
Schematic representation of Raman spectroscopy. After the sample (molecule) is exposed to an intense beam of monochromatic light in the frequency range of visible, near-infrared or near-ultraviolet region, most of the scattered light is at the same wavelength as the laser source (Rayleigh scattering). In contrast, a small amount of light is scattered at different wavelengths depending on the chemical structure of the analyte (Raman scattering). A Raman spectrum is a vibrational spectrum, where each peak corresponds to a specific molecular bond vibration, showing the intensity and wavelength position of the Raman scattered light.

As previously stated, clear surgical margins and a maximal degree of tumour resection strongly impact patients' outcomes. Raman spectroscopy has emerged as a promising biochemical technique to perform a non-invasive, real-time, automated, and *in vivo* assessment in different types of cancer (45, 46). The principle behind Raman spectroscopy is the interaction of light with the chemical bonds within a material or tissue. The term “Raman spectrum” refers to a distinct chemical fingerprint of a tissue's current biological composition and activity that can be used to identify the tissue or distinguish it from others. Differences in the biochemical compositions (fatty-acid concentration, collagen content, DNA/RNA concentrations) of malignant tissues and healthy ones lead to the generation of different spectral signatures, with peaks associated with elevated concentrations of DNA, RNA, and peri-nuclear proteins in tumour sites (Figure 2) (47, 48).

Hale Wills et al. used Raman spectroscopy to analyse fresh NB specimens and other paediatric neural crest tumours. In their study, they collected 862 fresh and 252 frozen specimens from different samples (normal adrenal glands, NBs, ganglioneuromas, nerve sheath tumours, and pheochromocytomas) to compare the spectra of the frozen tissue against fresh tissue, and the results demonstrated a strong correlation between the two. They were able to distinguish between pathologic conditions and normal adrenal tissue with 100% sensitivity and specificity (49). In 2012, Leslie et al. evaluated the use of Raman spectroscopy to diagnose paediatric brain tumours. They collected samples (fresh and frozen) of untreated paediatric medulloblastoma, gliomas, and normal brain samples and registered twelve Raman spectra per sample. The obtained spectra could accurately distinguish paediatric brain neoplasms from the normal brain tissue. Even within the same type of tumour, Raman spectroscopy differentiated high-grade ependymomas from low-grade ependymomas with 100% sensitivity and 96.0% specificity (50). More recently, Jabarkheel et al. presented their work on intraoperative detection of paediatric brain tumour margins. In detail, they investigated the potential for Raman spectroscopy to rapidly detect paediatric brain tumour margins with intraoperative images of fresh *ex vivo* paediatric brain tissue samples. All imaged samples underwent formal final histopathological analysis. They created a labelled Raman spectra dataset of paediatric brain tumours. Then they developed an end-to-end machine learning model to predict the final histopathology diagnosis from the spectral data, suggesting that machine learning approaches can be used to classify tumours and detect their margins (51).

As every tissue has distinct molecular fingerprint that can be used to differentiate it from other tissues, Raman spectroscopy has potentially endless applications. Creating a database for evaluating rare tumours and pathological conditions based on their spectrum might allow for a rapid, non-invasive, real-time diagnosis, with no need for tissue destruction, processing, or staining.

## High-Definition Cameras

### Short-Wave Infrared Camera

To date, biomedical fluorescence imaging has mainly relied on NIR-I (wavelength: 700–900 nm) dyes, which has been favoured over visible light (wavelength: 380–800 nm) due to less tissue autofluorescence and absorbance (34). However, the low tissue penetration, the high background noise and limited tissue contrast of NIR-I dyes have limited their surgical translations in children. In this respect, more recent studies are investigating short-wave infrared (SWIR, wavelength: 1,000–2,000 nm) fluorophores and cameras as promising tools for achieving higher contrast, greater sensitivity and improved penetration depths (52).

Compared to NIR-I molecules, SWIR technologies have negligible autofluorescence, reduced optical scatter, and lower or comparable absorption. These features improve spatial and contrast resolutions, particularly when imaging fluorescence below the tissue's surface (53). Although SWIR technologies are at a very early stage of development, they are proving to have a great potential that could promote their translation into the clinic.

Even if no SWIR dyes have been approved for clinical use in humans yet, the discovery that some NIR-I dyes (such as ICG) display bright emission tails over 1,000 nm offers exciting opportunities for enhanced surgical imaging, especially in the field of surgical oncology (34).

Hu et al. described using a multispectral imaging system able to cover a spectrum range from 400 to 1,700 nm, which allows the generation of SWIR, NIR-I, and visible light imaging. In particular, the authors used this optical-imaging instrument for aiding the FGS resection of primary and metastatic liver tumours in adults. Their results showed that SWIR images provided a higher tumour-detection sensitivity, a higher tumour-to-liver signal ratio, and an enhanced tumour-detection rate than NIR-I images (54). In another study, Suo et al. reported the design and the construction of a SWIR fluorescence endoscopy imaging system compatible with most current clinic endoscopies, which was used to image animal models of colorectal cancer with ICG conjugated bevacizumab (Bev-ICG). SWIR *in vivo* images of a subcutaneous colorectal model showed a specific accumulation of Bev-ICG with high contrast, proving its potential as a promising contrast agent for non-invasive imaging of tumours overexpressing VEGF. Moreover, the use of the imaging system for imaging a rat orthotopic colorectal cancer model showed that the SWIR endoscope provided an accurate real-time delineation of colorectal tumour, highlighting the potential of SWIR imaging in the field of endoscopic (55).

As mentioned previously, SWIR fluorescence imaging is at a very early stage of development, but it shows great potential for *in vivo* detection of tumours *in vivo*. Although the literature found was based solely on adult series, the application of SWIR devices to paediatric instrumentation would easily translate into the use of these technologies in paediatric cancer surgery. Moreover, the use of specific targeting by conjugating SWIR molecules with tumours' markers is of particular interest as it would combine the advantages of SWIR imaging (reduced autofluorescence, deeper penetration and reduced scattering) with the selective targeting of tumour cells.

### Radio-Guided Surgery Using Gamma Detection Probes

The concept of radio-guided surgery refers to the intraoperative detection of radionuclides using a radiation detection probe system. Intraoperative radiation probes can be divided into two main categories: gamma probes, which detect photon radiation (gamma rays or X-rays); and beta probes, which detect beta radiation (positrons or negatrons). There is a wide range of designs for small portable gamma cameras, which have been developed for intraoperative use (Table 2) (72). The use of radio-guided surgery in oncology provides real-time and specific visualisation of the extent of the disease, allowing the surgeon to assess surgical resection margins and to minimise the surgical invasivenes (72).

**Table 2:** Summary of characteristics and performances of small gamma cameras.

**Gamma camera**	**Detector**	**FOV**	**Size detector head**	**Energy (KeV)**	**Energy resolution (FWHM)**	**^**99m**^Tc sensitivity**
CarollReS (56–58)	Gd2SiO5 (Ce) PS-PMT	50 × 50 mm	78 × 78 × 275 mm		45%, ^57^Co	1,000 cps/MBq (theoretical)
eZ-SCOPE® (59)	CdZnTe	32 × 32 mm	60 × 60 × 220 mm	71–364	8.6%, ^99m^Tc	184 cps/MBq
GE camera (60, 61)	CdZnTe	40 × 40 mm	Height 150 mm	40–200	8%, ^99m^Tc	100 cps/MBq
Imaging probe (62)	CsI (Tl) PS-PMT	49 × 49 mm			20%, ^99m^Tc	210 cps/MBq
LumaGEM® (63)	CsI (Na) PS-PMT	20 × 20 mm		30–300	>20%, ^99m^Tc	
MediProbe (64, 65)	CdTe	14.08 × 14.08 mm	200 × 70 × 30 mm			6.5–33 cps/MBq (5 cm source-to-aperture distance)
Minicam®	CdTe	49 × 49 mm	Φ 95 mm height 150 mm	20–200		
Minicamll®	CdTe	40 × 40 mm	70 × 170 × 250 mm	30–300	5–7%, ^99m^Tc	
POCI (66)	YAP (Ce), IPSD	Φ 24 mm		Tc-99m, I-125, In-111	38%, ^57^Co	200 cps/MBq
Second POCI (67)	CsI (Na) IPSD	Φ 40 mm	Φ 95 mm height 90 mm	105–175	32%, ^99m^Tc	290 cps/MBq
Sentinella 102® (68)	CsI (Na) PS-PMT	40 × 40 mm	8 × 9 × 15 mm	50–200	15.9%, ^99m^Tc	90–900 cps/MBq (1 cm source-to-aperture distance) 27–72 cps/MBq (10 cm source-to-aperture distance)
SSGC clinical-type (69)	CdTe	44.8 × 44.8 mm	82 × 86 × 205 mm	Max 550	6.9%, ^99m^Tc	150 cps/MBq (high-resolution collimator) ramya1,600 cps/MBq (high-sensitivity collimator)
SSGC proto-type (70, 71)	CdTe	44.8 × 44.8 mm	152 × 166 × 65 mm	Max 550	7.8%, ^99m^Tc	300 cps/MBq

*FOV, field of view; FWHM, full-width half maximum of the ^99m^Tc (140 KeV) or 57Co (122 KeV) photopeak; SSGC, Small CdTe Gamma Camera; Gd2SiO5 (Ce) PS-PMT, Gadolinium oxyorthsilicate doped with cerium coupled with position sensitive photomultiplier tube; CdZnTe, cadmium zinc telluride; CsI (Tl) PS-PMT, Thallium doped cesium iodide coupled with coupled with position sensitive photomultiplier tube; CsI (Na) PS-PMT, sodium-doped cesium iodide coupled with position sensitive photomultiplier tube; CdTe, cadmium telluride; YAP (Ce), IPSD, Cerium doped Yttrium Aluminium Perovskite coupled with intensified position-sensitive diode; cps, counts per second*.

The use of gamma detection probes in the paediatric population is limited in the literature. Martinez et al. were the first to report the use of radio-guided surgery in 6 paediatric patients with stage II/IV NB. In their study, an intravenous injection of ^125^I-Tyr3-octreotide was given between 1.8 and 7.5 h before surgery. Out of the 17 ^125^I-Tyr3- octreotide binding sites found by the detection probe, 15 sites contained NB, which were not detected by standard intraoperative palpation and visualisation (73). The largest cohort of radio-guided procedures was published by Martinelli et al., who used either ^123^I-MIBG (injected 24 48 h before surgery) or ^125^I-MIBG (injected 3–5 days before surgery) for the intraoperative localisation of NB with a gamma detection probe. The probe was considered helpful in a vast majority of the cases. It permitted the detection of small, non-palpable, and difficult to access tumours and helped the surgeons define more accurately tumour margins (74).

Recently, there has been an increasing interest in developing hybrid gamma cameras (HGC) that provides the surgeon with fused optical and gamma images. Lees et al. described the first combined gamma and NIR fluorescence imaging producing dual-modality images in both laboratory simulations and the clinic. They reported the results of a patient's thyroid during the clinical investigation, showing that despite there was a reduction of the spatial resolution in the gamma image, due to the image processing performed before combining it with the optical image, there was an enhancement of the visual appearance when fused with its optical counterpart (75).

At the moment, there is no commercially available system offering hybrid imaging for clinical use. However, developing such systems has great potential both for cancer diagnosis and treatment, offering the surgeon the possibility of using a combination of probes to visualise different targets, improving the overall sensitivity and specificity of detection.

## Intra-Operative Loco-Regional Treatments

The introduction of novel adjuvant treatment, and their potential to clear microscopic residual of the disease, can help consolidate the loco-regional control reducing the risk of recurrence and progression of the disease. Photodynamic therapy (PDT), for example, can be used as treatment, salvage therapy, and palliative care for different tumours. However, PDT is, in general, a non-selective treatment acting on both healthy and cancerous cells (76). This limit can be overcome by using near-infrared photoimmunotherapy (NIR-PIT) that selectively kill cancer cells due to the conjugation of a special fluorophore with a monoclonal antibody. This conjugate will spare healthy tissues and vital organs, not causing any local side effects. Another innovative treatment is hyperthermic intraperitoneal chemotherapy (HIPEC), consisting of the intraperitoneal administration of heated chemotherapy treatment after cytoreductive surgery (77, 78).

### Photodynamic Therapy

**Figure 3:**
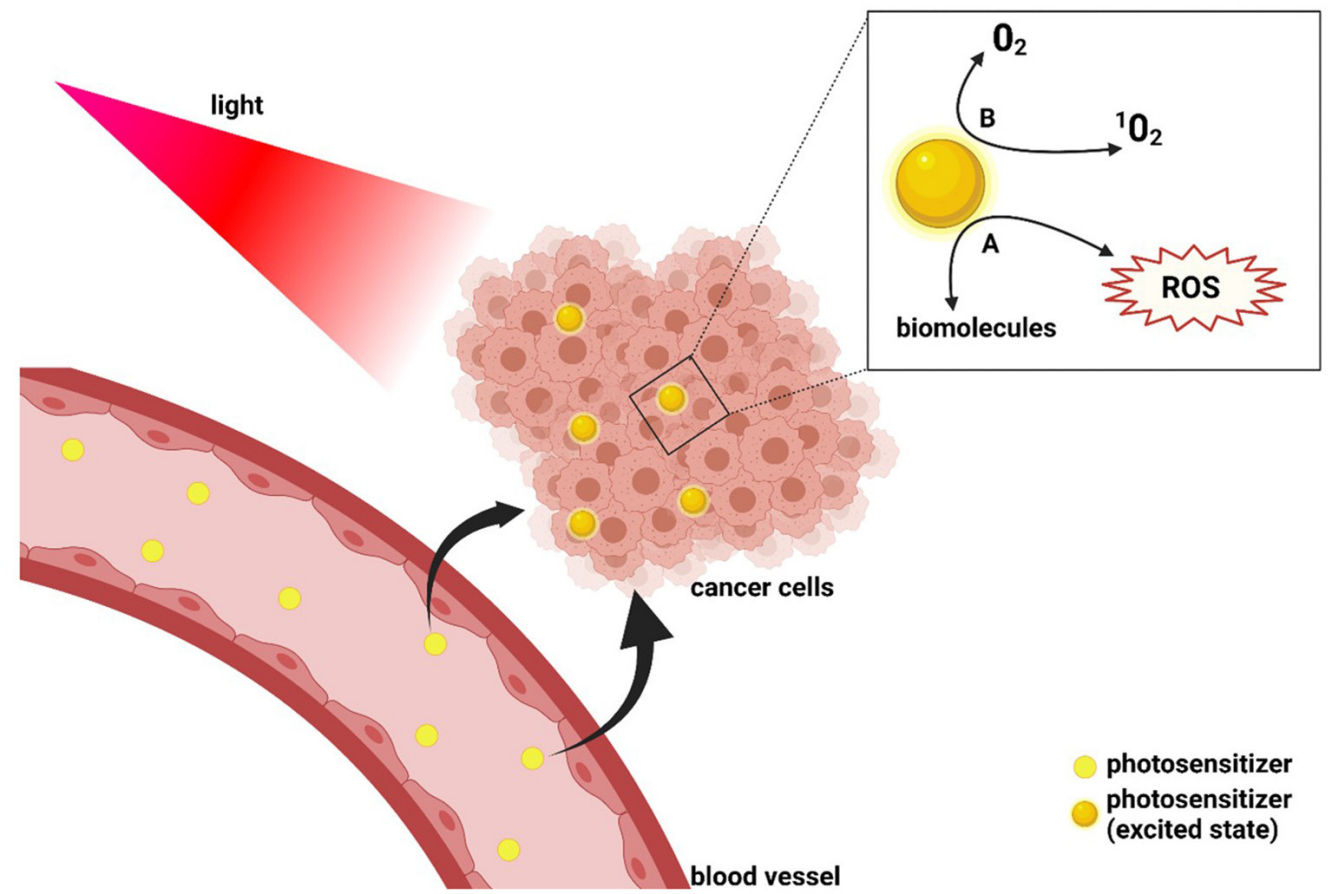
Schematic representation of photodynamic therapy mechanism of function. After administration, the photosensitive agent is irradiated at a wavelength that matches its absorption properties. The excitation of the photosensitiser leads to two different types of reaction: the production of reactive oxygen species (ROS) as a result of the interaction of the photosensitiser with biomolecules (Reaction A) and the generation of singlet oxygen (Reaction B).

From the wide range of therapies available in oncologic patients, PDT represents a novel adjuvant treatment capable of consolidating loco-regional control, aiming to reduce metastatic spread and progression of the tumour. The principle of PDT is a photochemical reaction between a photosensitive molecule (photosensitiser), light and molecular oxygen (76, 79). Administration of PDT starts with the intravenous, intraperitoneal or topical administration of a photosensitiser, followed by light exposure, which leads to the creation of radicals (Figure 3) (80).

The adult literature explored the role of PDT in tumours' treatment such as breast, brain, head, and neck gastrointestinal and genitourinary system tumours (81–85). Endoscopic procedures with PDT in oesophageal cancer showed less morbidity and mortality than surgery, even though neoplastic recurrence may be higher (81, 86). The application of PDT also appears to be significantly correlated with more prolonged survival in advanced cholangiocarcinoma. A study by Gonzalez-Carmona et al. showed that the combination of PDT and chemotherapy resulted in significantly longer overall survival than either PDT or chemotherapy alone in patients with advanced unresectable cholangiocarcinoma (87). Moreover, although surgical resection remains the standard of care for non-small-cell lung cancer, PDT has been used intraoperatively after tumour debulking in a phase II clinical trial. This study showed an improvement of patients' survival with a 73.7% local control rate at 6 months and a median overall survival of 21.7 months, probably related to the eradication of microscopic residuals, which usually cause the recurrence of the disease (88).

### Near-Infrared Photoimmunotherapy

The previous results on PDT encourage a potential use of this technique also in paediatric oncology. However, PDT exerts a non-selective action against cells relying on reactive oxygen species (ROS) generation, NIR-PIT allows a selective killing of target cells by conjugating NIR light-absorbing dye (IRDye700DX) to a cancer-targeting moiety (such as a monoclonal antibody) The conjugate is injected intravenously and binds to the specific cancer cells expressing the target antigen on the cell membrane. When the conjugate is exposed to the NIR light (~690 nm), axial ligands of the IRDye700DX molecule dissociate from the main molecule causing the photoactivated chemical to change from a highly hydrophilic to a highly hydrophobic compound. This leads to the damage and rupture of the cellular membrane with micro-perforations, blebbing, and bursting, resulting in necrotic cell death (89). The rupture of the cell membrane leads to the release of tumour-specific antigens into the tumour microenvironment and promotes dendritic cell maturation, eliciting the host immune system against the dying tumour cells (90).

The literature on the use of NIR-PIT in the paediatric population is scant. Nouso et al. showed that the administration of anti-GD2-IRDye700DX followed by NIR-PIT significantly suppressed cell viability compared to an anti-GD2 monoclonal antibody. Thus, their results showed NIR-PIT as a promising anti-tumour strategy to enhance the therapeutic efficacy of anti-GD2 immunotherapy for high-risk NB (91). Maruoka et al. (92) hypothesised that the administration of IL-15 with cancer cell-targeted NIR-PIT could increase anti-tumour host immunity. Most of the findings regarding the use of NIR-PIT in treating tumours can be found in the preclinical adult literature, where its potential has been explored in different types of tumours, going from head and neck tumours to gastrointestinal, lung, and lung and gynaecological tumours (93–95).

Overall, NIR-PIT could offer several benefits, like its tumour-cell specificity with virtually no damage to adjacent healthy tissues and vital organs. The same conjugate might also be used intra-operatively to guide the surgeon in localising the tumour. Lastly, NIR-PIT relies on a form of non-ionising radiation, with no limits to its total cumulative dose, meaning that multiple cycles could be safely employed. Further clinical studies are needed to prove the therapeutic potential of NIR-PIT in improving loco-regional control of tumours with reduced risk of recurrence and an overall benefit on survival.

### Hyperthermic Intraperitoneal Chemotherapy

HIPEC is an innovative treatment that aims to treat cancers with deposits or involvement of the peritoneum. It consists of an infusion of a heated chemotherapeutic solution that circulates in the abdominal cavity for 60–120 min to maximise cancer cell killing. However, as the solution is not able to penetrate deep into tumours, the treatment must be performed after cytoreductive surgery to debulk the tumour from the abdominal cavity. When performed in specialised centres, HIPEC has a rate of side effects comparable to any other digestive surgery, including paralytic ileus, haemorrhages, infections, fistulas, abscesses, haematological toxicity, and kidney failure. While this technique is routinely used in adults, currently, there are no guidelines in the paediatric populations where its use remains to be determined (77).

In 2015 Hayes-Jordan et al. reported the first 50 cases treated in their institution in the USA with cytoreductive surgery and HIPEC. The age range of their cohort was from 3 to 21 years old and with a median follow-up of 21.9 months for the surviving patients. Patients diagnoses included desmoplastic small round cell tumours, rhabdomyosarcomas, mesotheliomas, and other carcinomas. The HIPEC was performed using a closed technique, and it was added to chemotherapy and radiotherapy treatment for all the patients who demonstrated a partial response to neoadjuvant chemotherapy. Patients with desmoplastic small round cell tumours also underwent postoperative total abdominal radiation and postoperative chemotherapy. The results of their study showed that patients who had a complete cytoreduction, who were then treated with HIPEC, had a reduced risk for recurrence than those who had an incomplete cytoreduction. Patients' outcome was also affected by the peritoneal cancer index, with patients with a significant tumour burden having a median overall survival lower than patients with a lower score (19.9 vs. 34 months). Overall, cytoreductive surgery and HIPEC proved to be a safe alternative for extensive or refractory abdominal tumours, with the best outcome experienced by patients with desmoplastic small round cell tumours and those with complete cytoreduction (78). The same group reported their experience with cytoreductive surgery and HIPEC on paediatric girls with ovarian carcinoma, diffuse peritoneal disease, and no disease outside the abdominal cavity. Eight girls, previously treated with chemotherapy and surgery, were included in the study (age range 3–18). Three out of the eight patients recurred and died, while the remaining patients remained disease-free from 2 to 6 years post-HIPEC. The cohort showed an overall survival of 64% and relapse-free survival of 62%. Despite the small size of this cohort of patients, complete surgical resection, Cytoreductive surgery (CRS) and HIPEC proved to be a valid alternative that should be considered in paediatric patients with diffuse peritoneal disease from ovarian origin (96).

Most recently, Gesche et al. published their results on the use of CRS and HIPEC in a cohort of 6 patients below the age of 5 with intraperitoneal rhabdomyosarcoma. After surgery, HIPEC was performed using a closed technique with the administration of cisplatin and doxorubicin for 60 min at 42.5°C. Chemotherapy solution was eliminated by repetitive irrigation of the abdominal cavity with ringer solution. A peri- and postoperative hydration protocol was used to reduce the risk of HIPEC-associated renal failure. Their results demonstrate the safety and feasibility of CRS and HIPEC in this young age group with only low-grade side effects and no grade 3 or grade 4 toxicities (77). Sjoberg Bexelius et al. published the first case of CRS and HIPEC in a paediatric centre in the UK at the beginning of this year. This case report is about a 7-year-old girl diagnosed with an abdominal desmoplastic round cell tumour with peritoneal and liver metastases. The little girl, who is currently in complete remission 4 months after treatment, underwent CRS combined with HIPEC after six cycles of chemotherapy, followed by whole abdominopelvic radiotherapy and maintenance chemotherapy for 12 months. Although the long-term survival advantage of this technique is still uncertain and its use in children with this condition remains uncertain, this study proposed HIPEC as a potential alternative to selected children in the UK (97).

The use of HIPEC requires a multidisciplinary collaboration between adult peritoneal malignancy services, paediatric oncology, paediatric surgery, and intensive care services. To prove its clinical benefit, further data are needed, mainly concerning the paediatric population under 12 years of age. Furthermore, as there is no standardised way of administering HIPEC with variations in chemotherapy protocols, doses, lengths of treatment, a uniform guideline should be provided to have more consistent results.

## Conclusions

Apart from robotic surgery and augmented reality, oncology surgery has not changed significantly in the last decades, especially for paediatric patients. However, the research in this field is rapidly evolving. The devices and technologies presented in this study have the potential to revolutionise surgical oncology through more effective visualisation and removal of cancer. Thus, the oncology surgeons of the future need to remain up to date with the vast range of bioengineering advances that will possibly reach clinical practise in the next few years. These technologies will improve the effectiveness of surgery, leading to significant benefits for the children we want to cure.

## Author Contributions

LP and SG contributed to the study conception and design. LP and IP took care of the data acquisition. Critical revision and final approval were provided by KC and SG. All the authors contributed to the drafting of the manuscript and approved the submitted version.

## Funding

We thank the Medical Research Council (Grant No. MR/T005491/1) and the Wellcome/EPSRC Centre for Interventional and Surgical Sciences at University College London (WEISS, Grant No. 203145Z/16/Z), who supported the study.

## Conflict of Interest

The authors declare that the research was conducted in the absence of any commercial or financial relationships that could be construed as a potential conflict of interest.

## Publisher's Note

All claims expressed in this article are solely those of the authors and do not necessarily represent those of their affiliated organizations, or those of the publisher, the editors and the reviewers. Any product that may be evaluated in this article, or claim that may be made by its manufacturer, is not guaranteed or endorsed by the publisher.
